# Methods of probing the interactions between small molecules and disordered proteins

**DOI:** 10.1007/s00018-017-2563-4

**Published:** 2017-06-19

**Authors:** Gabriella T. Heller, Francesco A. Aprile, Michele Vendruscolo

**Affiliations:** Department of Chemistry, University of Cambridge, Cambridge, CB2 1EW UK

**Keywords:** Disordered proteins, Small molecules, Drugs, Binding, Molecular interactions

## Abstract

It is generally recognized that a large fraction of the human proteome is made up of proteins that remain disordered in their native states. Despite the fact that such proteins play key biological roles and are involved in many major human diseases, they still represent challenging targets for drug discovery. A major bottleneck for the identification of compounds capable of interacting with these proteins and modulating their disease-promoting behaviour is the development of effective techniques to probe such interactions. The difficulties in carrying out binding measurements have resulted in a poor understanding of the mechanisms underlying these interactions. In order to facilitate further methodological advances, here we review the most commonly used techniques to probe three types of interactions involving small molecules: (1) those that disrupt functional interactions between disordered proteins; (2) those that inhibit the aberrant aggregation of disordered proteins, and (3) those that lead to binding disordered proteins in their monomeric states. In discussing these techniques, we also point out directions for future developments.

## Introduction

Disordered proteins do not adopt well-defined secondary and tertiary structures under native conditions [1–7]. These proteins can be represented as ensembles of many conformationally distinct states, each with its own statistical weight (i.e. its probability of being occupied) [1–7]. Quite generally, all proteins exhibit some level of disorder, ranging from those that have just short dynamic terminal regions to those that are almost completely unstructured [1–7]. In many cases, the conformational heterogeneity of the latter proteins is believed to play important biological roles, as it enables them to interact with myriad partners. This multifunctionality is further enhanced by structural variations from post-translational modifications, as well as by the presence of multiple isoforms as a result of alternative splicing or pre-translational modifications [8]. Consequently, disordered proteins and proteins with disordered regions can act as central hubs in protein interaction networks for crucial regulation and signalling processes [9, 10]. Thus, it is not surprising that the dysregulation of disordered proteins is often correlated with biochemical pathways involved in cancer, cardiovascular diseases, diabetes, autoimmune disorders, and neurodegenerative conditions [10–12]. Illustrative examples of the involvement of disordered proteins in disease include the Cip/Kip cell cycle inhibitors, breast cancer type 1 susceptibility protein, and securin in the case of cancer, amyloid β, tau, α-synuclein, and huntingtin in the case of neurodegenerative disorders, and amylin (IAPP) in the case of type II diabetes [13].

For both structured and disordered proteins, the molecular mechanisms underlying protein-associated diseases can be divided into two broad categories, as a pathological condition can be triggered by either the total or partial inactivation of a protein (loss of function) or the acquisition of a new aberrant activity (gain of function). A well-known example of loss-of-function mechanism involving a disordered protein is that of the tumour suppressor protein, p53. p53 is a multi-domain protein with extended unfolded regions under native conditions, including its N-terminal and C-terminal domains. Several cancer-related mutations of p53 are localized in these regions and alter the interactome of this protein, thereby inhibiting its regulatory activity [8, 14]. On the other hand, an example of a disordered protein that exhibits gain of toxic function in disease is α-synuclein. Several missense mutations and genomic multiplications of α-synuclein affect its native state, solubility and cellular interactions, eventually prompting the protein to form amyloid aggregates associated with Parkinson’s disease [15–22].

Despite the high prevalence of disordered proteins in diseases, it is still very challenging to target these proteins using therapeutic compounds [11, 12, 23–29]. Two major obstacles in the drug discovery process for disordered proteins are: (1) the limited number of fully quantitative experimental techniques that can accurately probe disordered protein interactions with candidate therapeutic molecules compared to those available for ordered proteins, and (2) a lack of understanding of the molecular mechanisms underlying such interactions.

**Fig. 1:**
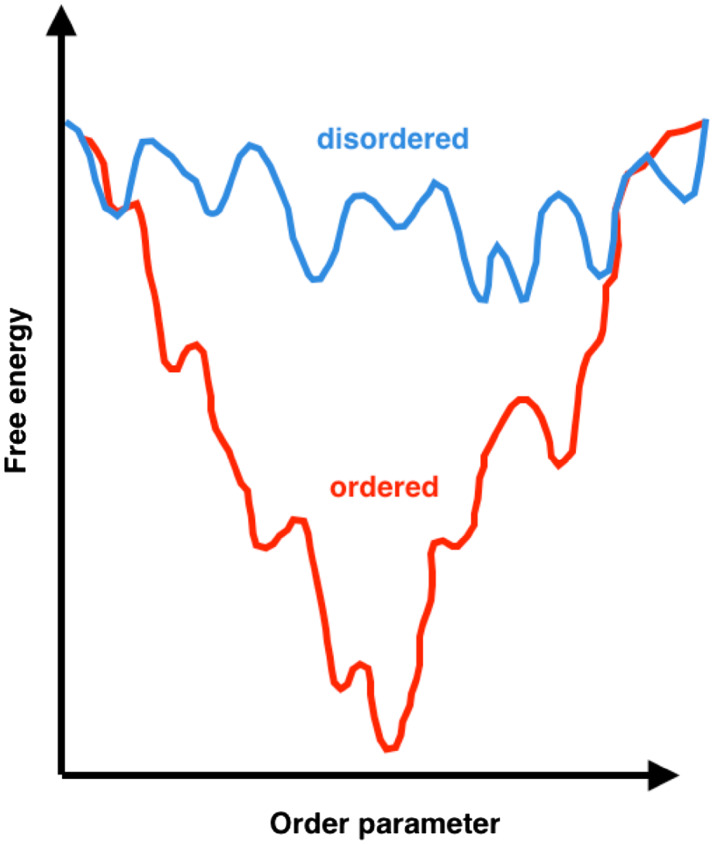
Schematic representation of the free-energy landscapes of ordered and disordered proteins. Structured or ordered proteins (*red*) have a free-energy landscape with a well-defined global minimal conformation, which can bind small molecules with high affinity. In contrast, disordered proteins have multiple minima within their free-energy landscape, which represent the many conformations capable of interacting with small molecules with lower affinities

For the past six decades, techniques such as X-ray crystallography have yielded highly accurate structural insights about ordered proteins, which have paved the way for drug discovery and potency-optimization efforts. Such techniques, however, are generally poorly informative in the case of disordered proteins as a result of their highly dynamical nature. Progress has been made when the disordered proteins are not fully disordered and have highly ordered regions or domains for which crystal structures can be obtained, as was done to identify small-molecule inhibitors of the cancer-associated p53-murine double minute 2 (MDM2) interaction [30, 31]. However, protein crystallization is largely inapplicable to the vast majority of disordered proteins due to their conformational heterogeneity. While ordered proteins usually have a single, well-defined conformation representing a global free-energy minimum with the potential to bind small molecules with high affinity, the free-energy landscape of disordered proteins is characterized by a large number of local minima (Fig. 1). These minima correspond to the many conformations within the structural ensemble populated by disordered proteins, which can transiently bind small molecules with very weak affinity. In fact, disordered proteins can remain disordered in their bound states, and characterization of these bound states is only recently becoming possible due to experimental and computational advances [32–35]. There are also examples of disordered proteins interacting with partner proteins in which they undergo disorder-to-order transitions through coupled folding upon binding mechanisms [36, 37] or templated-folding [38], resulting in high specificity, but low affinity complexes with usually large surface areas [37]. While these folded complexes may be crystallized, the identification of potential binding pockets from the crystal structures of the bound states is far from trivial [23]. Despite these challenges, several small molecules have been identified to modulate the behaviour of disordered proteins including the neurodegeneration-associated α-synuclein [39] and amyloid β [40], and the cancer-associated p27^Kip1^ [41], c-Myc [35, 42–44], and EWS–FLI1 [45].

To make further progress, effective techniques to probe small-molecule binding to disordered proteins must be further developed. Indeed, the lack of such methods has been a major bottleneck for the identification of molecules able to interact with disordered proteins and modulate their disease-promoting behaviour. The current situation has resulted in a poor understanding of the mechanisms underlying these interactions, which has, in turn, hindered the development of drugs active against disordered proteins. In this review, we highlight the most prominent techniques that have enabled so far major contributions to be made to the understanding of whether and how small molecules can alter the disease-promoting behaviour of disordered proteins.

## Methods of identifying inhibitors of interactions between disordered proteins

The lack of well-structured binding sites within disordered proteins makes it challenging to target them directly using well-established drug discovery techniques developed for ordered proteins such as enzymes and receptors. Instead, some approaches involve targeting disordered proteins indirectly, by blocking their binding interfaces with other proteins [46] and lipid membranes [47]. In the cases where the binding surfaces are structured and well characterized, this approach can be highly specific, as it may be amenable to standard affinity-optimization techniques. However, a thorough understanding of the binding partners involved, as well as the contact sites of interest, must usually already be well established. In this section, we discuss the state of art of this approach and present some notable examples of what we define here as ‘interface blockers’.

Perhaps one of the most well studied systems in this context is the interaction between the two disordered proteins, c-Myc and Max, which have been probed by a wide variety of techniques. c-Myc is a transcription factor associated with many types of cancer, whose interaction with its regulator Max is associated with cellular growth, metabolism, apoptosis and differentiation [48, 49]. A basic helix–loop–helix-leucine zipper (bHLHZip) in each of these two proteins facilitates their coupled folding and binding upon dimerization, creating an interface of approximately 3200 Å^2^ in the coiled-coil dimer [35, 43, 46, 50]. The identification and characterization of small-molecule inhibitors of this interaction has represented a major milestone in demonstrating the feasibility of therapeutic targeting of disordered proteins.

### Fluorescence resonance energy transfer

Inhibitors of the c-Myc/Max association were found via high-throughput screening assays of combinatorial small-molecule or peptidomimetic libraries based on fluorescence resonance energy transfer (FRET, Table 1) measurements of the bHLHZip domains of c-Myc and Max fused to cyan fluorescent protein (CFP) and yellow fluorescent protein (YFP), respectively [51–53].

**Table 1:** Summary of techniques discussed in this review

Technique	Applicability	Limitations	Throughput	Selected examples	Further reading
Fluorescence resonance energy transfer (FRET)	Detection of modulators of protein–protein interactions; detection of protein–ligand interactions	Fluorescent labels required	High	c-Myc/Max and ligands [51–53], protein-tyrosine phosphatase 1B and MSI-1436 [56]	[54]
Yeast two-hybrid system	Detection of modulators of protein–protein interactions	Indirectly quantitative	High	c-Myc/Max and ligands [44, 58]	[200]
Fluorescence polarization	Detection of modulators of protein–protein interactions; detection of protein–ligand interactions	Fluorescent labels required	High	c-Myc/Max and ligands [43], c-Myc-Max complex/DNA and ligands [59, 60]	[61–63]
Circular dichroism spectroscopy (CD)	Determination of the changes in secondary structure upon binding	Low sensitivity	Low	c-Myc/Max and ligands [35, 43]	[65, 201]
Fluorescence-based aggregation kinetic assays	Identification of inhibitors of protein aggregation	Fluorescent dyes required	High	Aβ [40], α-synuclein [47]	[89, 92, 97]
Surface-plasmon resonance/other surface-based techniques	Real-time detection of modulators of protein–protein/interactions; detection of protein–ligand interactions	Non-specific interactions may yield false positives	Medium	EWS-FLI1 and YK-4-279 [45]	[116, 117, 202]
Small-angle X-ray scattering (SAXS)	Detection of large conformational changes upon binding at nanometer resolution	Low resolution	Variable	Protein-tyrosine phosphatase 1B and trodusquemine [56]	[114, 115]
Thermal denaturation screening	Detection of monomeric binders	Non-quantitative	High	Nuclear protein 1 and ligands [119]	[203]
Isothermal titration calorimetry (ITC)	Label-free measurement of the heat associated with binding events	Significant heat change required upon binding	Medium–low	Nuclear protein 1 and ligands [119]	[121]
Single-molecule techniques	Determination of the structure and dynamics of disordered proteins in presence of ligands	Labels required	Medium–low	α-Synuclein [125, 129]	[125]
Mass spectrometry	Localization of noncovalent interactions	May miss ligand interactions, gas-phase dissociation constants may differ from solution	Low	Polycationic spermine and α-synuclein [141]	[133, 134, 204]
Nuclear magnetic resonance (NMR) spectroscopy	Detection of protein–ligand interactions at atomic resolution	Ligand monitoring: fast, protein monitoring: time intensive, isotopic labelling may be required	Medium–low	Osteopontin/heparin [169], protein-tyrosine phosphatase 1B and trodusquemine [56]	[145, 147]
Integrative structural biology methods	Modelling of unbound/bound structural ensembles	Time intensive, computationally expensive	Low	c-Myc and ligands [28, 34]	[6]

FRET signals arise upon the interaction of two chromophores, whereby an excited donor chromophore transfers its excitation energy to a nearby acceptor chromophore through nonradiative dipole–dipole coupling. This transfer of energy results in both a quenching of the fluorescence of the donor and an appearance of a fluorescence emission spectra of the acceptor. Importantly, the efficiency of the energy transfer is strongly dependent on the distance between the donor and acceptor (in the range between 1 and 10 nm), thereby enabling FRET to effectively quantify molecular associations [54].

In a seminal study, c-Myc/Max FRET experiments identified two compounds, called IIA4B20 and IIA6B17, capable of inhibiting c-Myc-dependent cell growth [51]. These molecules, however, also showed activity against another oncogenic transcription factor, c-Jun, suggesting poor specificity. Nevertheless, a follow-up study using a related combinatorial library with members assembled from a racemic, *trans*-3,4 dicarboxylic acid template yielded c-Myc/Max dimerization inhibitors that did not affect c-Jun [52], suggesting that specificity is potentially achievable in targeting disordered proteins.

Additional libraries have been screened against c-Myc/Max interfaces based on FRET experiments. One library consisted of 285 so-called ‘credit card’ compounds, designed to insert themselves into a shallow protein–protein interface hotspot of about 600 Å^2^ rich in hydrophobic and aromatic residues, and force the protein partners to remain in their monomeric forms [53]. After FRET screening, the initial hits were further characterized by an electrophoretic mobility shift assay (EMSA) to confirm their activity. Based on the observation that the electrophoretic mobility of a bound system is less than that of the unbound system, EMSA provides quantitative information about modifiers of DNA-binding protein complexes [55]. In general, compounds in the ‘credit card’ library tend to be planar, with varying chemical diversity, designed to have favourable enthalpic contributions from van der Waals interactions, π-stacking, and favourable entropy gains from desolvation [53]. Two compounds, NY2267 and NY2280, were identified to disrupt c-Myc–Max dimerization, and inhibit both specific DNA binding and its associated oncogenic transformation. As in the case of IIA4B20 and IIA6B17, however, these molecules also showed an inhibition of c-Jun [53].

FRET experiments have also been used to characterize the conformational changes induced when the natural product trodusquemine (also known as MSI-1436) allosterically inhibits protein-tyrosine phosphatase 1B (PTP1B) by interacting with its disordered region. By labelling the N- and C-termini of PTP1B with CFP and YFP, respectively, conformational changes upon binding could be detected, suggesting that the presence of trodusquemine induced a more compact structure upon binding [56]. This binding-induced conformational change was further characterized by nuclear magnetic resonance (NMR) spectroscopy and small-angle X-ray scattering (SAXS), discussed in the “Methods of characterizing ligand interactions with monomeric disordered proteins”.

### Yeast two-hybrid system

**Fig. 2:**
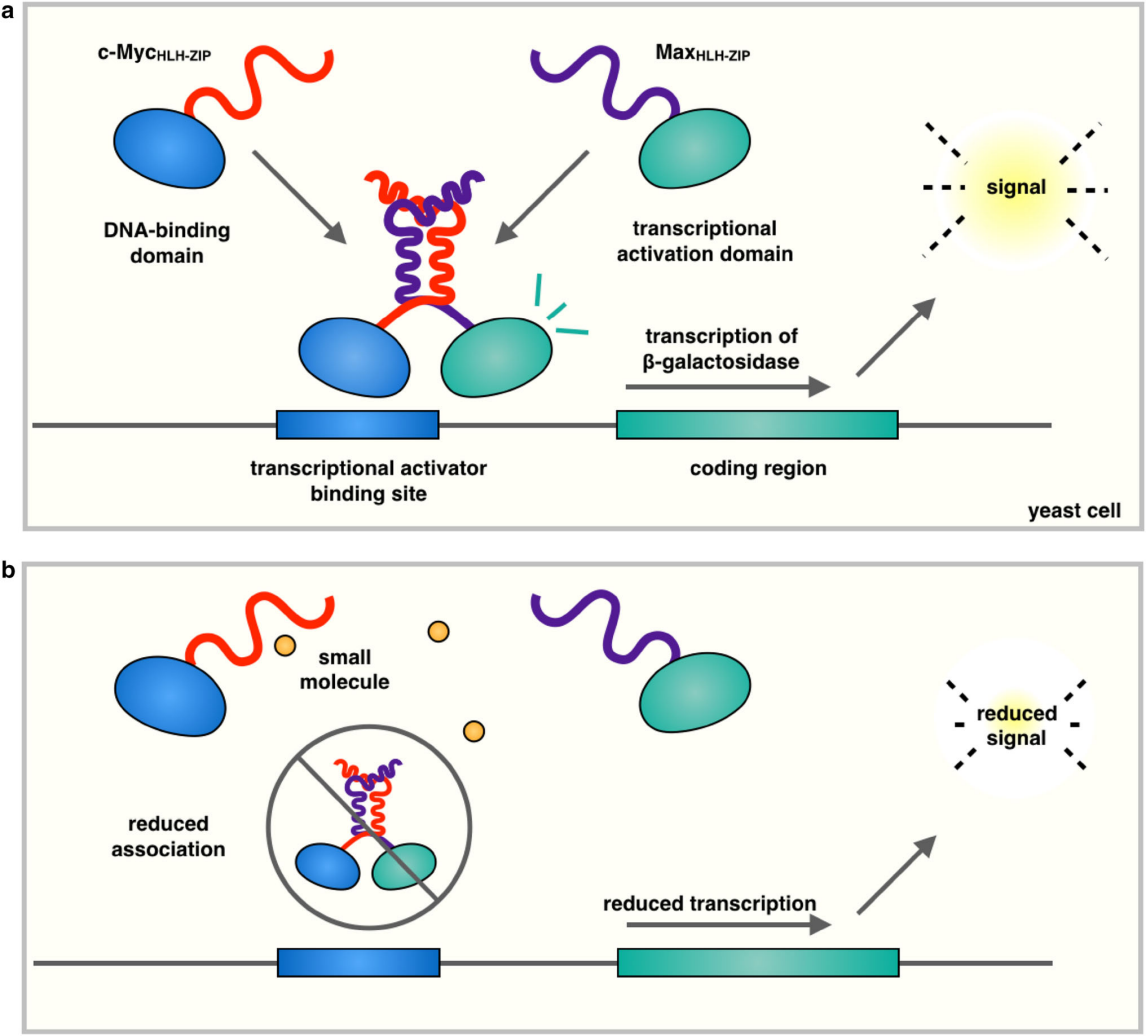
Schematic representation of the yeast two-hybrid system. In the type of yeast two-hybrid system used to identify inhibitors of c-Myc/Max dimerization [51–53], recombinant genes encoding the HLHZip domain of c-Myc fused to the DNA-binding domain and HLHZip domain of Max fused to the transcriptional activation domain are introduced into a yeast cell (**a**). Upon c-Myc/Max association, the transcriptional activation domain induces expression of β-galactosidase in a quantitative manner (**b**)

Another powerful high-throughput screening technique involves using a yeast two-hybrid system to identify small molecules capable of disrupting protein-protein interaction. In a yeast two-hybrid system (Table 1), two candidate interacting proteins are fused to the DNA-binding domain (BD) and the activation domain (AD) of a transcription factor, which hence functions only when a complex between the two proteins is formed. Using this setup the HLHZip domains of c-Myc and Max were fused to the BD and AD domains, respectively, of the yeast transcription factor Gal4. Only upon c-Myc/Max dimerization a fully functional transcriptional activator is produced, which is able to induce the expression of β-galactosidase from the corresponding gene containing a Gal4-binding site within its promoter [44] (Fig. 2). As small molecules that alter this association prevent the induction of β-galactosidase in a quantitative manner, yeast two-hybrid systems represent effective screening tools to identify interface blockers. We should add, however, that although high-throughput screening approaches of this type identified a number of c-Myc/Max dimerization inhibitors, screening against other similar dimers suggested that the majority of the resulting hits lacked specificity. Many of these hits, including the small molecule 10058-F4, were confirmed to directly interact with monomeric HLHZip region of c-Myc [35, 43]. The optimization of the 10058-F4 structure led to analogues with increased potency [42], and the development of a pharmacophore model [57]. A further discussion of the interaction of 10058-F4 with its c-Myc is continued below in the “Methods of characterizing ligand interactions with monomeric disordered proteins”. A yeast two-hybrid system was also used to identify dihydroxycapnellene, a coral-derived sesquiterpene capable of preventing c-Myc–/Max dimerization and effective against the proliferation of cancer cells [58].

### Fluorescence polarization

**Fig. 3:**
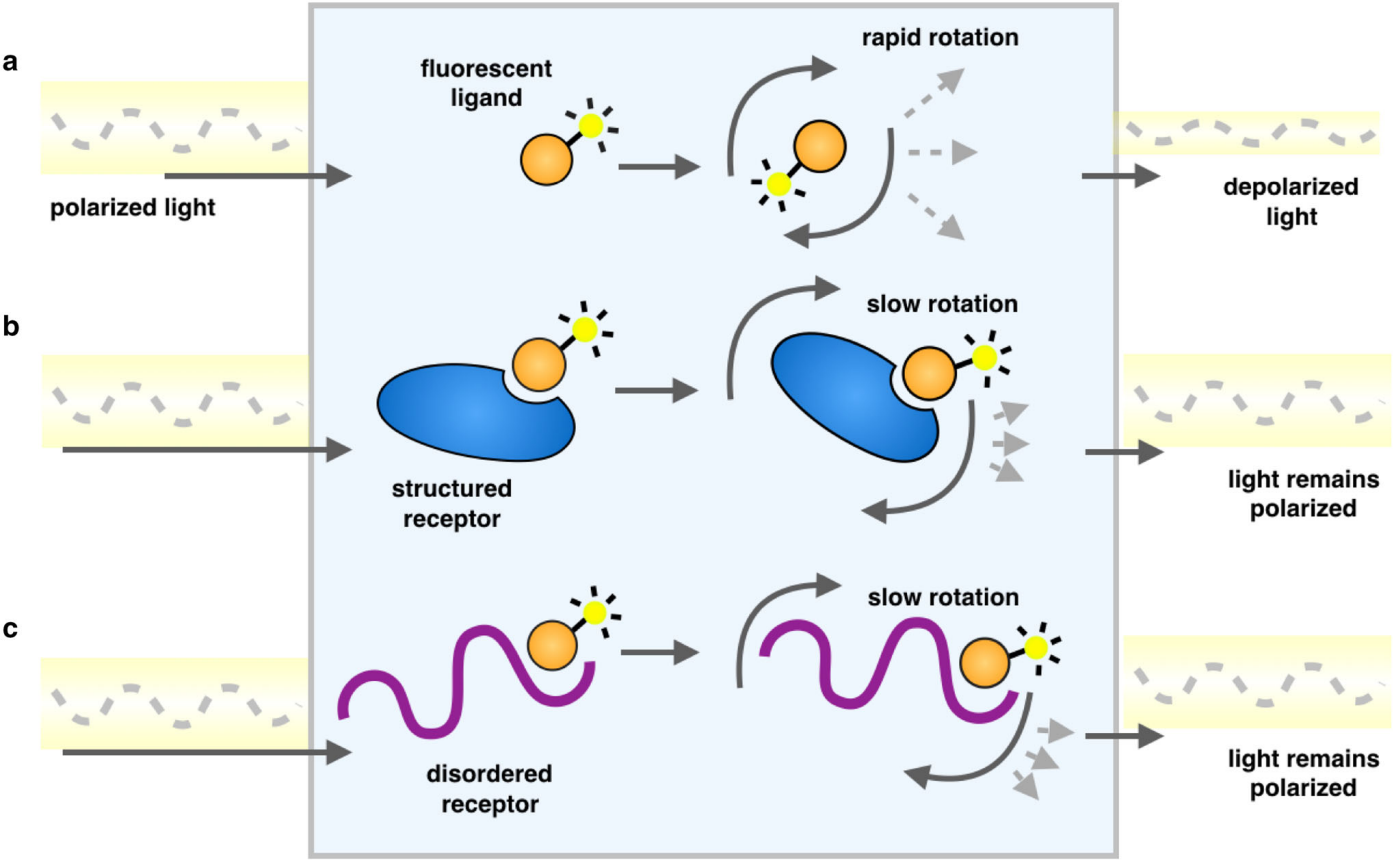
Schematic representation of a fluorescence polarization experiment. As a result of rapid tumbling of molecules in solution, when a fluorescently labelled ligand is excited with plane-polarized light, the resulting emitted light is largely depolarized (**a**). Upon binding another species, a larger proportion of the emitted light remains in the same plane as the excitation energy, because the rotation is slowed as the effective molecular size increases, whether it is an ordered molecular structure (**b**) or one that is disordered (**c**)

Further studies focused on targeting the interface between the c-Myc/Max dimer and DNA by screening molecular libraries based on fluorescence polarization experiments [59, 60] (Table 1). Fluorescence polarization is a physical phenomenon that occurs when fluorescent small molecules are excited with polarized light. The resulting emitted light is largely depolarized due to the rapid tumbling of the small molecules in solution. However, when the small molecules are bound to other species, this tumbling is quantitatively slowed as their effective hydrodynamic radii are increased, thereby better maintaining the polarization of the emitted light (Fig. 3). Because the measured polarization value is a weighted average of the free and bound states, fluorescence polarization is an important biophysical tool for drug discovery to measure the fraction of a fluorescent ligand bound to a receptor [61–63].

Fluorescence polarization screens identified two compounds, Mycro1 and Mycro2, capable of preventing DNA binding of the c-Myc/Max dimer and transcription, measured by a c-Myc reporter gene. This approach was implemented by labelling the DNA target sequences with fluorophores. However, these molecules showed signs of non-specificity as they also inhibited Max/Max DNA binding in addition to transcription from an AP-1 dependent reporter [59]. Upon further screening of Mycro1 and Mycro2 derivatives, Mycro3 was identified to strongly inhibit c-Myc transcription while leaving AP-1 unaffected [60]. Furthermore, once the binding sites of 10058-F4, as well as that of the compound 10074-G5, within the c-Myc monomer were established using deletion and mutagenesis studies of the bHLHZip domain of c-Myc, fluorescence polarization competition affinity experiments were performed to determine the binding sites of seven other inhibitors, taking advantage of the intrinsic fluorescence of the drug-like molecules. Six of these seven compounds bound one of the binding sites already established, whereas one, 10074-A4, bound a region adjacent to the site of 10075-G5. It is notable that these three binding sites are all within a span of 85 residues, which suggests that drug-binding regions may fall within specific disordered sequences [43].

### Circular dichroism spectroscopy

The case of targeting monomeric c-Myc demonstrated the feasibility of targeting a disordered protein in its monomeric state as a therapeutic strategy [25, 35, 44, 64]. Many different optical techniques contributed to the characterization of this type of binding interaction, including in particular circular dichroism (CD) experiments (Table 1), which are based on the differential absorption of left- and right-handed circularly polarized light and can be used to determine the secondary-structure content of proteins. With this approach it was demonstrated that 10058-F4 and 10074-G5 caused an unfolding of the c-Myc/Max coiled-coil dimer into disordered monomeric states. Furthermore, CD was used to confirm the two distinct 11 and 19-residue binding regions identified for 10058-F4 and 10074-G5, respectively, by deletion and mutagenesis studies. This binding was further characterized by performing fluorescence polarization titrations, which take advantage of the intrinsic fluorescence of these compounds [35, 43]. In addition to conventional CD experiments, CD spectroscopy obtained using beamline synchrotron radiation offers improved sensitivity at a wider range of wavelengths to detect subtle changes upon complex formation [65].

### Byproducts of screenings to identify enzyme inhibitors

Small molecules have also been identified to interact with the Alzheimer’s-related disordered amyloid-β peptide (Aβ, discussed more in detail below). Serendipitously, some of these small molecules were not identified by direct screening against the peptide itself, but rather during a search for modulators of γ-secretase, which, together with β-secretase, cleaves the amyloid precursor protein (APP) to produce toxic Aβ. Derivatives of two modulators (tarenflurbil and fenofibrate) were created to contain a benzophenone group (a UV-active moiety used for labelling) and a biotin tag. It was thus found that these derivatives bind directly to APP within the Aβ region, and act as a ‘molecular clamps’ or substrate-targeted inhibitors preventing the cleavage of Aβ [66].

## Chemical kinetics approaches to identify protein aggregation inhibitors

Under certain conditions, some disordered peptides and proteins, such as Aβ, α-synuclein and amylin, undergo a self-assembly process, which leads to the formation of fibrillar aggregates known as amyloid fibrils. This aggregation process is typically associated with pathological conditions such as Alzheimer’s and Parkinson’s diseases, and type II diabetes [15, 67–69]. Given the clinical relevance of the aggregation phenomenon, efforts have been put forward to inhibit the aggregation process from occurring, many of which have been carried out via in vitro assays [23, 70–77].

The kinetics of formation of these aggregates can be monitored experimentally via the use of amyloid-specific fluorescent dyes (Table 1), such as the thioflavin T (ThT). Complementary biophysical techniques to monitor this process include transmission electron microscopy (TEM), atomic force microscopy (AFM), and Fourier transform infrared spectroscopy (FTIR). Such experiments highlight the presence of three typical macroscopic phases of aggregation in vitro, namely, the lag phase, growth phase, and plateau phase. The molecular pathways that control this aggregation process, however, have been extremely difficult to characterize, mainly because of the challenges in establishing accurate and highly reproducible in vitro assays for monitoring fibril formation and in formulating an overall kinetic theory to analyse the resulting measurements. For example, as the aggregation of Aβ has emerged as a key feature of the onset and progression of Alzheimer’s disease [78, 79], various compounds [80–87] have been reported to interfere with the aggregation process of Aβ, but none of these molecules has yet found a therapeutic application because of the poor understanding of their mechanism of action.

Recently, this situation has begun to change due to advances in defining a chemical kinetics theory of aggregation [15, 88, 89]. It is now understood that the overall aggregation process is the result of complex non-linear combinations of microscopic events, including: (1) primary nucleation, in which initial aggregates form from monomeric species; (2) elongation, in which existing fibrils increase in length by monomer addition, (3) secondary nucleation, whereby the surfaces of existing aggregates catalyse the formation of new aggregates and (4) fragmentation in which existing fibrils break apart, increasing the total number of fibrils [15, 88]. The contributions of each of these microscopic events to the lag, growth, and plateau phases are highly protein and condition specific. It has thus become possible to obtain microscopic rates from macroscopic measurements, thereby revealing the mechanisms of aggregation of specific proteins and the effects of small molecules on such mechanisms [15, 88, 89].

Furthermore, reproducible protocols to measure the kinetics of Aβ aggregation have also been established [88, 90–92], thus providing accurate data that could be fitted with the chemical kinetics theory. These advances have helped in elucidate the crucial mechanisms in the aggregation process of Aβ42, the 42-residue form of Aβ, which forms the most toxic species associated with Alzheimer’s disease. In particular, it has been found that once a critical concentration of amyloid fibrils has formed, secondary nucleation overtakes primary nucleation in becoming the major source of toxic oligomers [93]. Further developments of this chemical kinetics framework have shown that therapeutic strategies against amyloid aggregation should not simply aim at a complete inhibition of fibril formation, but rather at specifically targeting toxic oligomeric species, as generic and non-specific effects could lead to the increase in the concentration of these oligomers and hence result in a negative outcome in terms of suppressing pathogenicity [89].

**Fig. 4:**
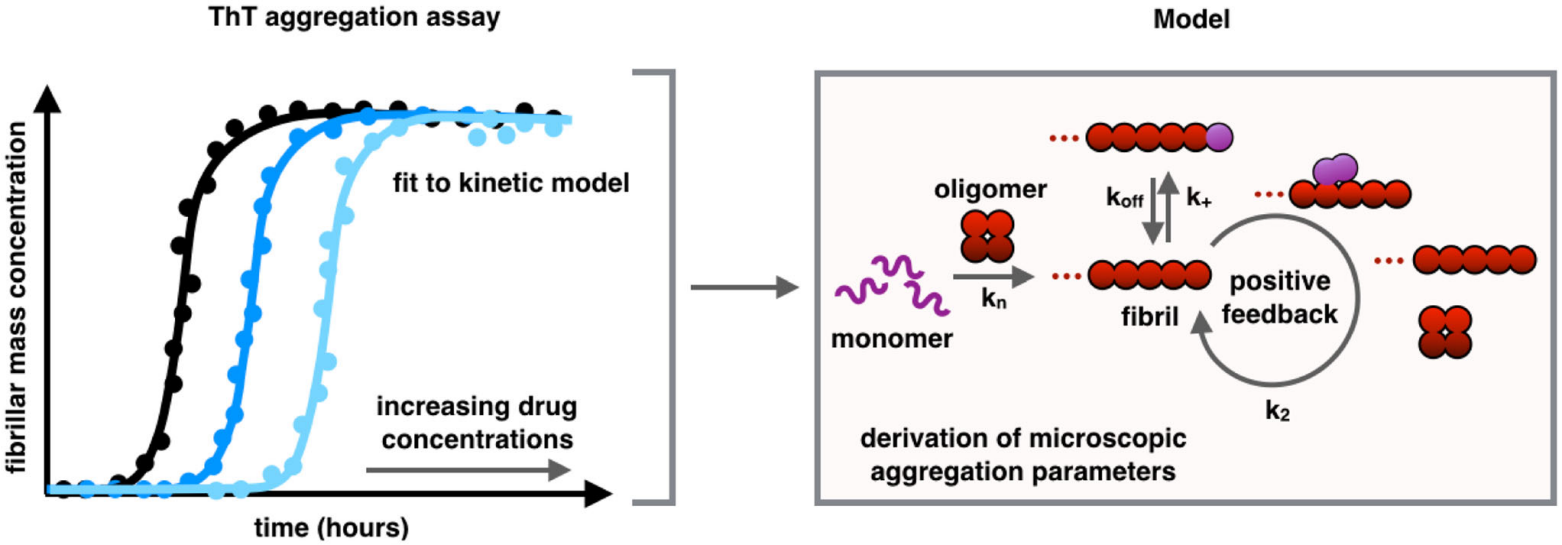
Schematic representation of a fluorescence-based kinetic aggregation assay. Aggregation assays to monitor the kinetics of formation of fibrillar aggregates are performed using a fluorescence dye molecule, in this case thioflavin T (ThT). Binding can be fitted with a kinetic model from which microscopic aggregation parameters can be derived [88, 91, 92]. Monitoring how these microscopic parameters change in the presence of small molecules is a powerful approach for screening molecules capable of inhibiting the aggregation process [40, 89]

Recently, the small molecule bexarotene was discovered to target the primary nucleation step in the aggregation of Aβ42. Its presence delays the formation of oligomers of Aβ42 and suppresses the toxicity in neuroblastoma cells and in a *Caenorhabditis elegans* model of Aβ-mediated dysfunction. While this small molecule suggests that compounds may be found that act as 'neurostatins' to delay Alzheimer’s disease if taken before the onset of disease, research efforts are now also focused on developing a strategy for specifically targeting secondary nucleation processes which may yield a therapeutic capable of inhibiting toxicity after the onset of symptoms [40]. As a proof-of-principle, it has been demonstrated that the molecular chaperone Brichos is able to block the formation of toxic oligomers of Aβ42 by specifically inhibiting the secondary nucleation [94, 95]. To this end, kinetic analysis applied to a range of derivatives of bexarotene has been recently employed in order to evolve systematically potential inhibitors and to obtain libraries of compounds with increased anti-aggregation activity [96] (Fig. 4).

In contrast to monitoring aggregation with fibril-specific dyes, an alternative in cell high-throughput screening method for detecting Aβ inhibitors has been proposed which involves the expression of a fusion of Aβ42 to the green fluorescent protein (GFP) in *Escherichia coli* cells. In the absence of inhibition, the aggregation of Aβ42 results in a quenching of the GFP fluorescence. However, in the presence of an aggregation inhibitor, the fluorescence of GFP is preserved, thus enabling the identification of molecules based on a triazine scaffold that inhibit Aβ aggregation [97].

Furthermore, in addition to small-molecule compounds, protein-like compounds capable of specifically suppressing protein aggregation have inspired new technological advances aimed to produce peptides, such as β-hairpins [98] and β-breakers [99, 100], antibodies [101], antibody fragments [102, 103], or other biomolecules, including molecular chaperones [104], to act as highly effective and specific protein aggregation inhibitors. Specifically, antibody fragments, particularly single-domain and single-chain antibodies, are becoming highly explored molecules for the inhibition of amyloid aggregation. Since the first production of conformationally distinct antibodies able to uniquely target fibrillar and oligomeric species of various amyloidogenic proteins [105], many other amyloid-specific antibodies have been generated by means of direct immunization or using hybridoma technology [101], phage display [106] or, more recently by rational design [99, 103].

In addition to directly modulating homogeneous aggregation processes, as illustrated above in the case of bexarotene for Aβ aggregation, small molecules have also been shown to also impact heterogeneous nucleation processes associated with aggregation. For example, the antimicrobial aminosterol, squalamine, alters the heterogeneous aggregation of α-synuclein [47]. The primary nucleation of α-synuclein is an intrinsically slow process, whose rate increases by a thousand fold as a consequence of the interaction of α-synuclein monomers with lipid membranes [107]. Squalamine has been proved to inhibit the lipid-induced primary nucleation of α-synuclein by displacing monomers from the membranes [47].

In summary, as the cases of the Aβ and α-synuclein have shown, reproducibility of high-throughput fluorescence aggregation assays and a chemical kinetic framework underlying these complex aggregation processes have emerged as essential tools to identify molecules as modulators of these toxic aggregation processes. Furthermore, these tools enable the quantification of the effects of such therapeutics on various microscopic aggregation steps, thus creating novel opportunities in drug discovery against neurodegenerative diseases.

## Methods of characterizing ligand interactions with monomeric disordered proteins

### Experimental methods to characterize the binding of molecules to disordered proteins in their monomeric forms

In contrast to targeting disordered proteins in their aggregated or bound forms, it is often desirable to target them in their monomeric forms, upstream of any biological effect. Small molecule binding to a monomeric disordered protein, however, may come at a high entropic cost due to restraining a conformationally heterogeneous protein into a bound state [11]. Consequently, disordered protein interactions with small molecules are not readily amenable to the traditional ‘binding site docking’, which is generally exploited in the case of designing and optimizing small-molecule binders of structured proteins. Even some mechanisms used to describe protein–protein interactions involving at least one disordered partner tend to not be applicable because generally in these cases, since the enthalpic contributions over large interaction surface areas outweigh the entropic costs. In the case of small molecules, which lack these large surface areas, the entropic cost of restraining a disordered protein can be too high. Instead, the currently reported interactions between small molecules and monomeric disordered proteins are relatively weaker than traditional drug–protein interactions [2], may involve multiple binding sites, and the protein may remain disordered in its bound state [108].

X-ray crystallography is the gold standard for determining small-molecule binding sites within ordered proteins for which an average conformation is well defined at the atomic level by mapping corresponding electron densities to atomic coordinates [109]. In the case of disordered proteins, however, dynamical regions generally appear as missing electron density [110–113]. Therefore, solution-state techniques that do not require crystallization, such as nuclear magnetic resonance (NMR) spectroscopy and other techniques described here (Table 1), coupled within integrative structural biology methods (Table 1) are better suited to probe disordered proteins, as they can directly characterize their conformational heterogeneity.

### Small-angle X-ray scattering

Small-angle X-ray scattering (SAXS, Table 1) is a label-free biophysical technique that is particularly well suited to quantitatively analyse heterogeneous and flexible systems such as disordered proteins in solution [114]. Based on the scattering of X-rays upon exposure to a sample, it is a useful technique to quantify conformational changes upon ligand binding [115]. As previously mentioned, SAXS, in combination with FRET and NMR experiments, was employed to demonstrate the compaction of PTP1B upon binding trodusquemine, which alters the allosteric communication of the disordered C-terminal region of PTP1B and the folded catalytic domain, thereby inhibiting its phosphatase activity [56].

### Surface plasmon resonance and other surface-based techniques

Surface plasmon resonance (SPR, Table 1) is a sensitive, label-free, optical method based on the detection of the changes upon binding of the refractive index at the surface of a bio-functionalized gold-coated prism. At certain angles of incidence, electrons at the gold surface absorb some photons of the incident light, giving rise to surface plasmons. Because this phenomenon is extremely sensitive to changes in the surface of the biochip due to changes in mass, SPR is particularly sensitive for monitoring association and dissociation of biomolecules immobilized on a surface. SPR was used to screen a 3000-molecule library for small molecules able to bind EWS–FLI1, a predominantly disordered oncogenic fusion protein associated with Ewing’s sarcoma family tumours [45]. An initial hit was optimized to produce the small molecule YK-4-279, with a reported affinity of 10 μM, which showed in vitro and in vivo inhibition of the RNA helicase A binding ability of EWS–FLI1. Like SPR, other surface-based techniques including bio-layer interferometry (BLI) [116] and quartz crystal microbalance (QCM) [117] are extremely sensitive and well suited to study disordered protein interactions with small molecules [25]. We also point out, however, that with any surface-based technique, one should carefully minimize any non-specific interaction with the sensor or the tip, or to account for them appropriately in the analysis [118].

### Thermal denaturation screening

By comparing temperature-dependent denaturation patterns of proteins in the presence and absence of small molecules, one can identify potential hits, because interacting ligands may induce structural rearrangements and changes in stability (Table 1). These effects can also be monitored extrinsically using dyes, such as 8-anilino-1-naphthalene sulfonic acid (ANS), whose fluorescence increases upon binding to hydrophobic protein regions. This screening method was recently exploited to identify several binders, including trifluoperazine, of nuclear protein 1 (NUPR1) [119], which is of great therapeutic interest due to its association with pancreatic adenocarcinoma, and many other diseases [120]. However, one shortcoming of this approach is that there is no direct correlation between the stabilization effect and the affinity, making it difficult to rank hits.

### Isothermal titration calorimetry

Isothermal titration calorimetry (ITC, Table 1) is an experimental technique that measures the heat exchanged during binding events between molecules in solution [121]. In this experiment, direct measurements of the absorbed or released heat are taken as one binding partner (either the protein or ligand) is titrated into a solution containing the second binding partner, offering invaluable information that cannot be readily observed by other means. In one single experiment, one can obtain the binding constant (*K*_d_), Gibbs free energy of binding (∆*G*), enthalpy (∆*H*), entropy (∆*S*), and stoichiometry of the interaction. Furthermore, ITC has many advantages over other techniques; measurements can be carried out in a physiologically relevant buffer, no surface effects need to be taken into account, and the species of interest do not need to be immobilized or labelled [121]. In a standard setup, one binding partner, whose concentration is known, is titrated into a solution of the second binding partner, whose concentration is also known, while changes in the heat of the system are monitored. Over time, the protein–ligand system reaches equilibrium while the differences between heat changes diminish. Plotting the heats of the titration as a function of the molar ratio of ligand and protein inside the cell yields a curve that can be analysed with a binding model to determine the thermodynamic parameters [121].

In the case of disordered proteins, ITC can be particularly useful when a protein adopts a rigid conformation upon binding a partner, such that the contributions of enthalpy to the Gibbs free energy are significant. Such contributions can arise from the formation and breaking of noncovalent bonds, namely protein-solvent hydrogen bonds, protein–ligand bonds, van der Waals interactions, salt bridges, reorganization of atoms and solvent molecules near the binding site, and many more. ITC enabled a validation and quantitative ranking of the binders of NURP1 (introduced above in the “Thermal denaturation screening”) in terms of affinity, and suggested that this binding is largely entropically driven [119].

### Single-molecule methods

Major technological advances have recently created exciting opportunities to probe disordered protein interactions with ligands at the single-molecule level. Single-molecule techniques (Table 1) are particularly promising to probe the structure and function of disordered proteins, because measurements are not ensemble-averaged as in the case of the vast majority of other available experimental techniques. Generally, two types of these experiments can be performed to elucidate the interactions of disordered proteins with binding partners: fluorescence-based techniques [122, 123] and force-probe methods [124].

Single-molecule FRET measurements are one of the several fluorescence experiments that can be performed at the single-molecule level. Similarly to the bulk-phase FRET experiments (described above), single-molecule FRET techniques require labelling with donor and acceptor dyes, but both the dyes are generally on the same protein. Experiments can either be performed on surface-immobilized samples using a total internal reflection fluorescence (TIRF) setup or performed on freely diffusing molecules. While TIRF may enable the collection of long measurements of the fluctuations of a single molecule, interactions with the surface can perturb the native ensemble of the disordered protein. Consequently, it is more common to perform experiments on freely diffusing disordered proteins in which a laser is focused at a dilute solution (usually 50–100 pM) of labelled protein. The resulting fluorescence from both the donor and acceptor is measured and related to the distance between the two fluorophores, thereby reflecting the conformation of that molecule in the presence or absence of a ligand. Unlike bulk FRET measurements, this value is not ensemble-averaged, and many measurements enable one to construct the distribution of conformations within a given sample [125]. For example, single-molecule FRET was applied to study the conformations and dynamics of monomeric α-synuclein in the presence of sodium dodecyl sulphate (SDS) as a lipid mimetic. This technique enabled a detailed thermodynamic characterization of the multi-state conformational changes of α-synuclein folding in the presence of SDS [126].

Single-molecule force-probe microscopy also offers intriguing complementary approaches to the single-molecule fluorescence-based methods. These techniques involve the use of optical tweezers, magnetic tweezers, or atomic force microscopy by which the ends of individual protein molecules are constrained in order to apply and measure forces which yield information about their extensions and resulting conformational transitions [127]. This type of approach has been widely employed for characterizing the conformational and dynamic behaviour of disordered proteins, including α-synuclein [128, 129] and Aβ [130]. Furthermore, these techniques have characterized disordered and unfolded proteins in the presence of binding partners, including molecular chaperones [131] and ions [132].

### Mass spectrometry methods

**Fig. 5:**
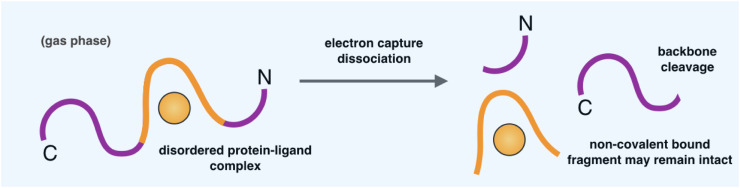
Schematic representation of mass spectrometry with electron capture dissociation (ECD). This is a technique that enables the identification of local binding regions within disordered proteins. ECD breaks covalent backbone bonds of the disordered protein, while leaving noncovalent interactions intact, thus preserving the disordered protein–ligand interaction [141]

The development of soft ionization methods, such as electrospray ionization (ESI) and matrix-assisted laser desorption/ionization (MALDI), has facilitated the application of mass spectrometry (MS) to protein characterization and protein binding, offering insight on stoichiometry, reversibility, specificity, and binding affinities [133, 134]. Furthermore, MS is a particularly powerful probe of protein behaviour due to its ability to monitor discrete conformers in a mixture [135–137]. While there are a number of MS-based techniques to probe protein behaviour, ranging from those that monitor how variance in buffer conditions affect the distribution of charge states into the gas phase [138, 139], to those that trap protein ions in the gas phase and observe conformational changes on a μs–s timescale [140], here we highlight one particular advance that has enabled the localization of a ligand binding site within a disordered protein. By combining ESI-MS with electron capture dissociation (EDC), a technique to fragment gas-phase ions (Fig. 5), the polycationic compound spermine was found to bind α-synuclein in the region of residues 106–138. It was shown that EDC breaks certain covalent backbone bonds of α-synuclein, while leaving noncovalent interactions intact, thus preserving the spermine–α-synuclein interaction [141]. This technique is highly complementary to nuclear magnetic resonance spectroscopy (see below) and other biophysical measurements, although it should be noted that the parameters observed in the gas phase may differ from those in solution, as the hydrophobic effect is essentially lost in the gas phase, while electrostatic interactions are strengthened due to the fact that the dielectric constant is lower than water [142, 143]. We also mention that MS methods have been applied to identify inhibitors of aggregation (introduced above) [134, 144].

### Nuclear magnetic resonance spectroscopy

Nuclear magnetic resonance spectroscopy (NMR, Table 1) can be employed in two complementary ways to monitor the binding between a disordered protein and a small molecule. Changes in the one-dimensional hydrogen spectrum of the ligand in the presence of a disordered protein offer a fast and sensitive indication of binding, but offers little insight regarding the binding site and mode of interaction. In addition, monitoring the protein (which usually requires ^15^N or ^13^C isotopic labelling) is a powerful method that can yield informative structural and dynamical binding information about disordered proteins, as a result of a systematic series of advances within the past decade [145–148]. In particular, the sensitivity of the latter technique offers highly quantitative insights into how the properties of disordered proteins change in the presence of small molecules. Quite generally, in NMR structural information is derived by exploiting the conformational dependence of the transitions between different energy levels of atomic nuclear spins, which can be made to split in an external magnetic field and resonate using electromagnetic radiation. While, in contrast with structured proteins, nuclear Overhauser effects (NOEs) [148] cannot always be readily exploited to obtain inter-proton distances for disordered proteins due to their conformational heterogeneity, other NMR parameters, including chemical shifts, hydrogen exchange rates, residual dipolar couplings (RDCs) and paramagnetic relaxation enhancements (PREs), can provide atomic-resolution structural information [145, 147, 149–151].

In contrast to the high-resolution assignments for globular proteins, which can be obtained using triple resonance coherence transfer experiments on isotopically labelled proteins, equivalent measurements of disordered proteins often yield overlapping peaks within collapsed spectra. This is a result of a combination of structural disorder and solvent exposure, which creates similar environments for many residues. This problem is often worsened by the low sequence complexity found within disordered proteins [145, 147, 152, 153], especially as they are enriched in proline residues, which are invisible to hydrogen-detected NMR spectra [154, 155]. Furthermore, such high solvent exposure also contributes to decreasing the signal-to-noise ratios for disordered proteins, as significant chemical exchange with bulk solvent reduces the intensities of amide hydrogen signals. While signal overlap of disordered proteins can be partially ameliorated by sample preparation at low pH and by taking measurements at low temperatures, the largest improvements have been a result of technological advances. Such advances include increased instrumental sensitivity, faster sampling rates exploiting longitudinal relaxation enhancements [156] and the use of non-uniform sampling for high-dimensionality experiments [145, 152, 153, 157, 158]. Additionally, by replacing hydrogen detection with carbon detection and by exploiting cryoprobe technology, it is possible to separate peaks accurately, while remaining insensitive to broadening and salt concentrations [147, 152, 153, 159]. Despite their poor spectral resolution, disordered proteins produce particularly sharp peaks, making them ideal for relaxation experiments, and as such, additional improvements include relaxation-optimized detection schemes [145, 160]. Furthermore, the structural properties of the aggregates formed by some disordered proteins can be studied by other NMR techniques such as solid-state magic-angle spinning which is discussed in detail elsewhere [161, 162].

**Fig. 6:**
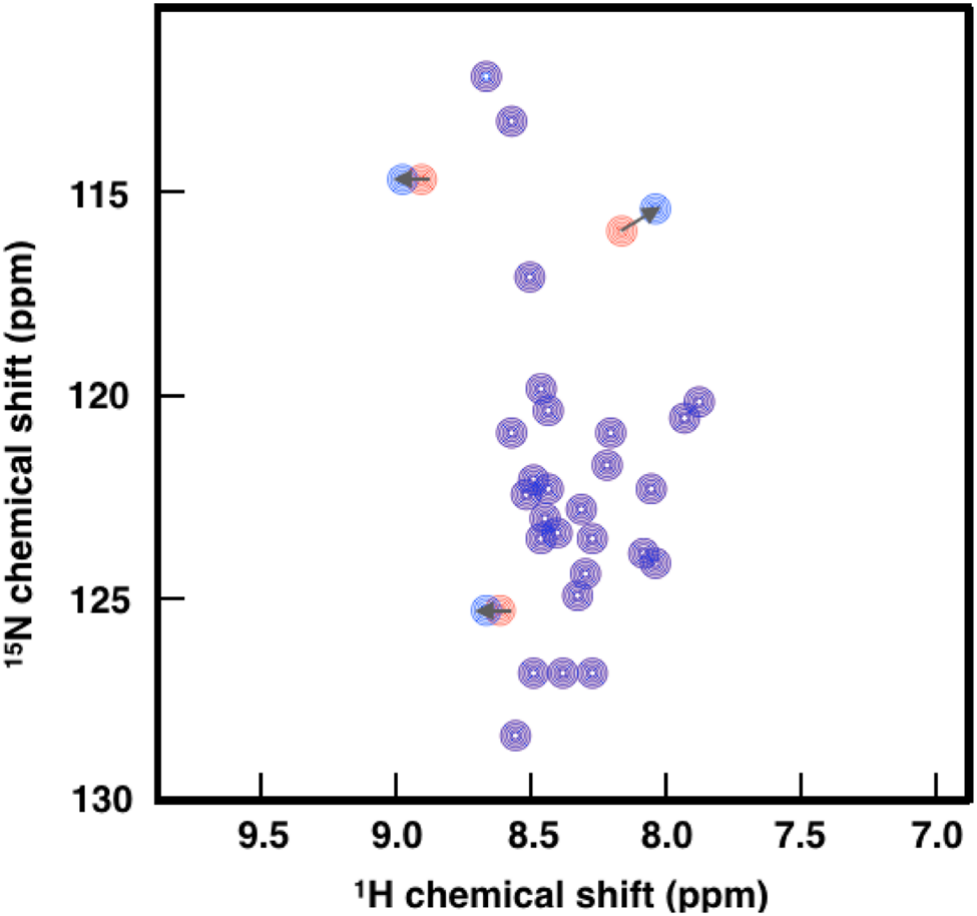
Schematic illustration of the chemical shift perturbation mapping method. By identifying and quantifying changes in two-dimensional spectra (in this case ^1^H–^15^N HSQC) in the absence (*red*) and presence (*blue*) of ligands, chemical shift perturbation mapping is a powerful technique to identify whether ligands interact with disordered proteins, and identify binding sites or locations of conformational change

Among the most useful NMR parameters for characterizing disordered proteins that we discuss here are chemical shifts, which report on the population-weighted average across the conformations sampled within a millisecond time scale. By calculating deviations from random coil values, one can describe the local geometry and quantify local secondary structure propensity in disordered proteins [163–165] and quantify changes in the absence and presence of therapeutic molecules. Furthermore, 2D NMR ‘fingerprint’ spectra, most commonly obtained with either ^1^H detected ^1^H–^15^N (HN) [166] and ^13^C detected ^13^C′–^15^N (CON) [167] for disordered proteins, are simple indicators of monomeric binding, and provide highly detailed site-specific information if protein assignments have already been established. While the HN experiment has higher sensitivity and requires less time to record, the CON experiment displays better spectral resolution, can detect proline residues, and is not prone to hydrogen-exchange-induced line broadening, and thus spectra can be recorded at higher pH and temperatures [159]. Chemical shift perturbations are a sensitive technique that can simultaneously provide binding affinity (*K*_d_) values and insight about a binding site or the location of conformational changes induced upon binding (Fig. 6). Finally, within integrative methods (see below), chemical shifts can be used to determine structural ensembles of disordered proteins [168].

Two-dimensional (2D) ^1^H–^15^N heteronuclear single quantum coherence spectroscopy (HSQC) experiments were used to confirm the binding of heparin to the intrinsically disordered osteopontin [169], an extracellular structural protein associated with many pathological conditions, including autoimmune diseases [170], cancer metastasis [171], Crohn’s disease and ulcerative colitis [172], allergy and asthma [173], and muscle disease [174]. Chemical shift differences only at certain residues between the free and bound forms of osteopontin suggested a specific interaction, and enabled mapping of the binding site [169]. Similarly, 2D ^1^H–^15^N HSQC experiments were used to characterize the specific binding of hits from ‘fragment-like’ small-molecule hits against p27, a disordered cell cycle regulator protein. These hits were identified from 1D ^1^H WaterLOGSY [175] and standard transfer difference (STD) [176] NMR screening methods, and one molecule in particular, was shown to inhibit the Cdk2/cyclin A binding function of p27 by fluorescence anisotropy and 2D ^1^H–^15^N TROSY [41]. A similar approach based on 2D ^1^H-^15^N TROSY [160] measurements was used to characterize the binding site of trodusquemine to the disordered C-terminal region of PTP1B [56]. Modifications to the HN and CON spectra enable the detection of other observables including RDCs, PREs, cross-relaxation and cross-correlation rates, in addition to solvent exchange rates. All these observables describe the structure and dynamics of disordered proteins at atomic resolution and are sensitive to changes in the presence of small molecules.

As mentioned above, RDCs are additional sensitive NMR observables that are particularly well suited to study disordered proteins in their monomeric states. These observables arise when disordered protein samples are partially aligned in a magnetic field by preparing samples in anisotropic media, for example, in a liquid crystal [177], polyacrylamide gels [178], filamentous phages [179], or bicelles [180]. As a result of restricted overall reorientation in the presence of the anisotropic media and dynamic conformational averaging, non-zero RDCs are observed which reflect the weighted average conformation of the ensemble [180]. Additionally, chemically modifying the disordered protein of interest with covalently attached paramagnetic spin labels, one can observe PREs, which report on tertiary structure, and the distances and orientations with respect to the principal axes frame of the paramagnetic centre. As for chemical shifts, RDCs and PREs can be implemented as structural restraints for ensemble generation [181], which is discussed in the next section.

### Integrative methods to characterize the effects of small molecules on protein ensembles

It is becoming increasingly clear that disordered proteins often bind ligands in transient and delocalized manners, in which the disordered protein remains in a disordered state upon association [32–34, 108]. In this context, high-resolution characterizations of conformational ensembles of disordered proteins, and of the ways in which such ensembles change in the presence of therapeutic molecules, have the potential to yield both functional mechanistic details and insights towards drug optimization.

Unfortunately, however, such detailed descriptions are currently difficult to obtain because the dynamic nature of disordered proteins makes it challenging to acquire accurate experimental measurements, as well as to interpret them in terms of structural models [5]. For example, as noted above, while NMR spectroscopy and other solution-state methods can provide valuable information on structural ensembles, these techniques alone are insufficient to provide all the conformational restraints needed to fully characterize the conformations within such ensembles. This is because experimental techniques, in addition to being inevitably affected by systematic and random errors, often measure sparse and sometimes ambiguous time- and ensemble-averages over the many heterogeneous conformations of the disordered proteins [6, 182].

To overcome these problems, computational techniques such as molecular dynamics simulations can provide accurate descriptions of protein ensembles [6]. In these simulations, the conformational space of a protein is sampled via the integration of the equations of motion over a sufficiently long time interval to ensure the exploration of the most relevant states and corresponding estimates of their populations. Such approaches have been used to investigate many small-molecule interactions with disordered proteins, particularly amyloidogenic ones [41, 83, 183–185] in addition to identifying potential binding pockets within disordered monomers [186]. Unfortunately, however, despite continuous advances, the force fields used to represent the interatomic forces needed to solve the equations of motion are still approximate [187–189], which leads to the need of validating the results through the comparison with experimental data [184, 186].

We should also remark that as most proteins of interest are large macromolecules in a complex environment, they are at the limit of what can be simulated. Conformational sampling as a result of limited computational resources is in fact often a major issue. While this problem can be partially alleviated through the use of enhanced sampling techniques [190, 191], the resulting ensembles may still be dependent on the simulation time, which is an approximation that requires careful control.

**Fig. 7:**
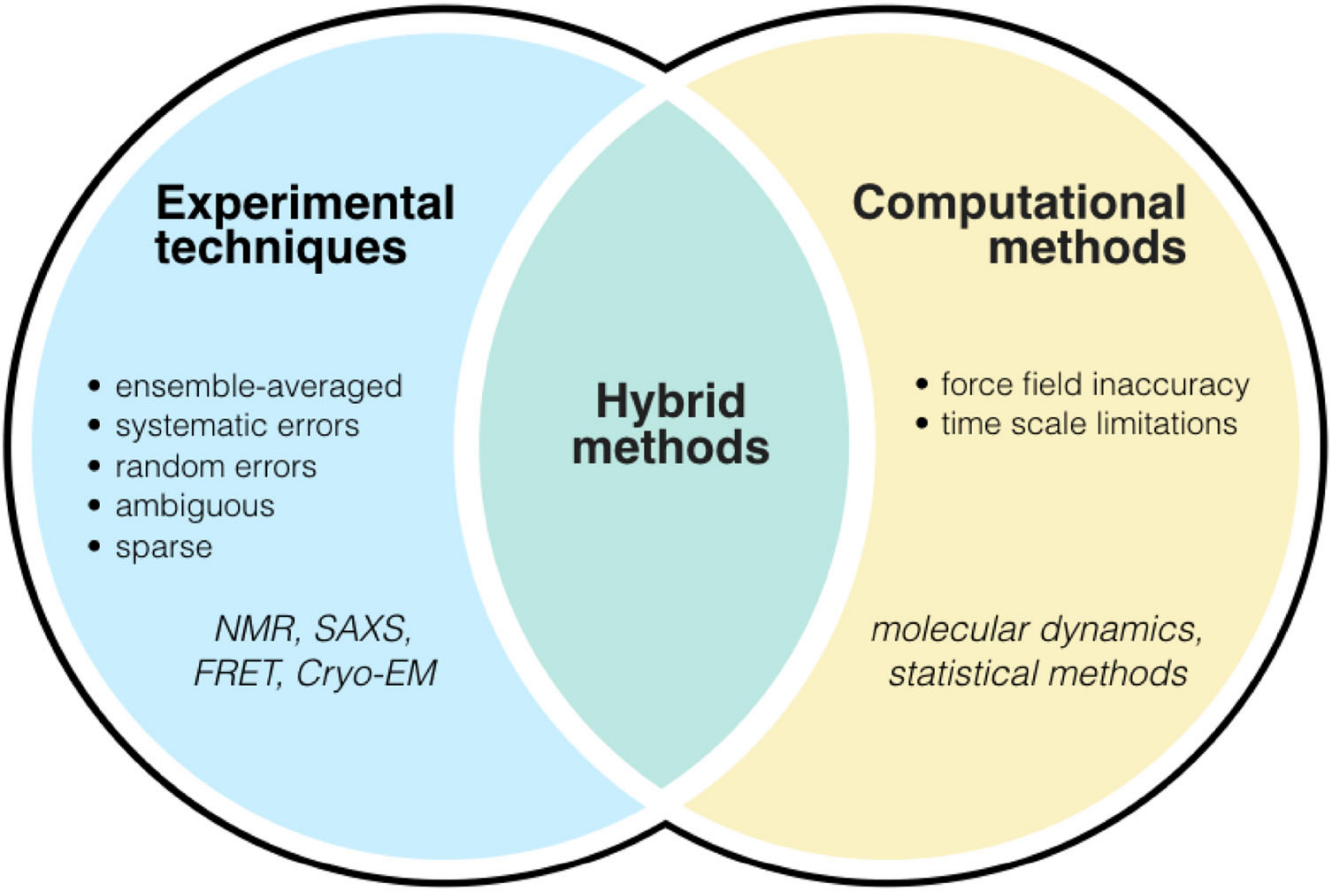
Schematic representation of integrative methods for protein ensemble generation. Integrative (or hybrid) methods, such as metainference [191, 199], combine the strengths of experimental techniques and computational methods to overcome the challenges associated with each technique alone [6]

We believe that an effective way forward is to bring together the advantages of the experimental and computational approaches (Fig. 7). A series of recent results indicate that by combining sparse experimental data on disordered proteins with a priori information from force fields [192–195] in molecular dynamics simulations, it is possible to generate descriptions of conformational ensembles with corresponding equilibrium probabilities for each state, such that the ensembles are consistent with the overall theoretical understanding of disordered proteins and with the available experimental data. There are many available methods for integrative structural ensemble determination of heterogeneous systems [6]. Because of the limited space that we have here, however, we only briefly highlight recent advances which enable one both to incorporate experimental data directly as structural restraints and to account for systematic and random errors by employing Bayesian inference techniques. These include ‘multi-state Bayesian modelling [196, 197], the ‘Bayesian ensemble refinement [198], and ‘metadynamic metainference’ [191, 199].

Integrative structural biology methods were used to characterize the binding interactions between binding sites within c-Myc and small molecules. Metadynamics simulations using NMR chemical shift data [35] as restraints were employed to show that these interactions are highly delocalized, the binding sites remain disordered, and the conformational space of the binding regions are slightly altered [28, 34].

In summary, integrative computational methods for determining ensembles of disordered proteins that incorporate experimental measurements and account for different sources of error represent a powerfully detailed and increasingly accurate approach to study the behaviour of ensembles in the presence of candidate therapeutic molecules.

## Conclusions and outlook

**Fig. 8:**
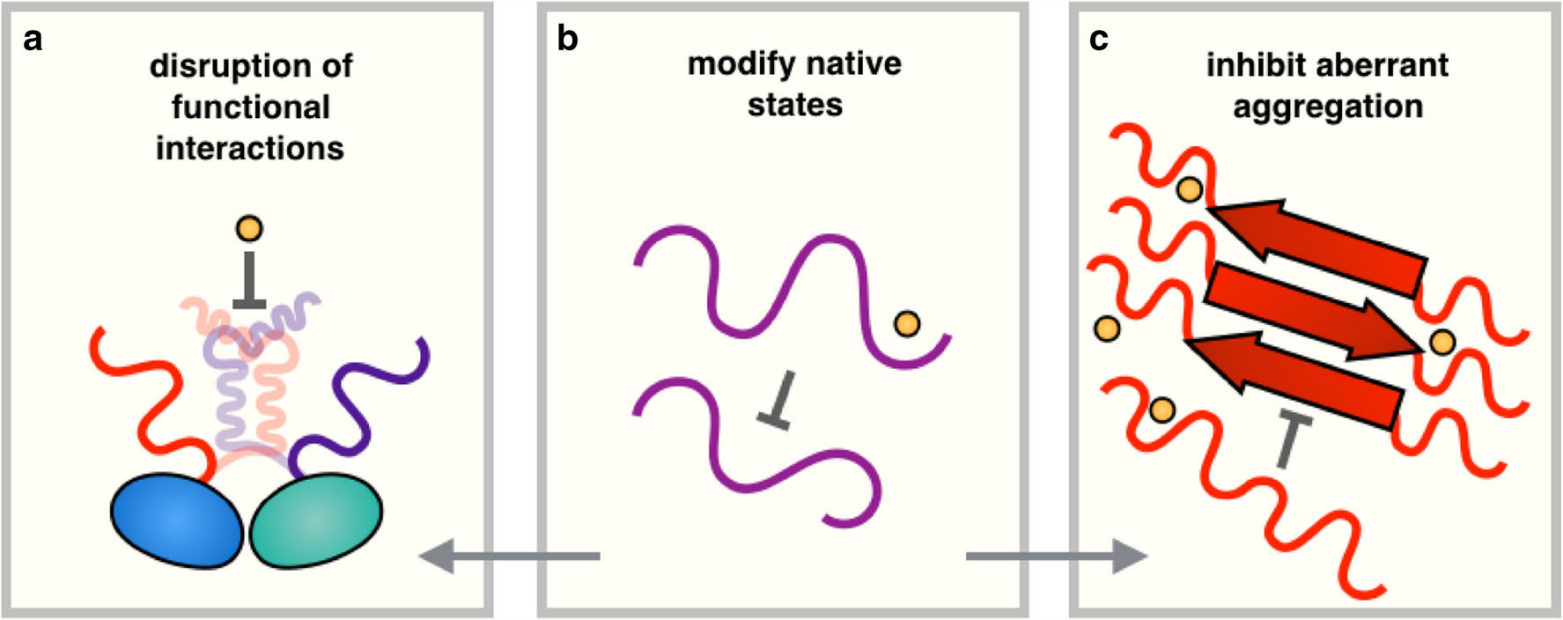
Summary of approaches for modulating the behaviour of disordered proteins using small molecules. Small molecules can be used to: **a** disrupt functional interactions, **b** modify the properties of native states, or **c** inhibit aberrant aggregation. Modifying the properties of monomeric disordered proteins (**b**) has the potential to also inhibit (**a**, **c**)

We have discussed three strategies to use small molecules to modify the behaviour of disordered proteins (Fig. 8). We have begun with the strategy of modulating the functional interactions involving disordered proteins using small-molecule inhibitors. We then reviewed recent advances in using chemical kinetics to identify compounds capable of blocking the aggregation of disordered proteins, and finally discussed methods of finding small molecules capable of binding disordered proteins, emphasizing the importance of more closely integrating experimental and computational techniques. Overall, we believe that upon further developments, the methods that we have reviewed will lead to a progressive ability to identify compounds of therapeutic interest for disordered proteins. We anticipate that an area of research of crucial importance will be to understand the role of specificity in these interactions, which will likely require the development of new assays, as well as possibly innovative conceptual tools.
